# Outcome and rational management of civilian gunshot injuries to the brain—retrospective analysis of patients treated at the Helsinki University Hospital from 2000 to 2012

**DOI:** 10.1007/s00701-019-03952-y

**Published:** 2019-05-25

**Authors:** Juhana Frösen, Oskari Frisk, Rahul Raj, Juha Hernesniemi, Erkki Tukiainen, Ian Barner-Rasmussen

**Affiliations:** 1 Department of Neurosurgery, Kuopio University Hospital and University of Eastern Finland, Kuopio, Finland; 2 Department of Plastic Surgery, HUS Helsinki University Hospital and University of Helsinki, Helsinki, Finland; 3 Department of Neurosurgery, HUS Helsinki University Hospital and University of Helsinki, Helsinki, Finland; 4 Juha Hernesniemi International Center for Neurosurgery, Henan Provincial People’s Hospital, Zhengzhou, China

**Keywords:** Gunshot wound, Brain injury, Mortality, Survival, Treatment indications

## Abstract

**Background:**

Treatment of gunshot wounds of the brain (GSWB) remains controversial and there is high variation in reported survival rates (from < 10 to > 90%) depending on the etiology and country. We retrospectively analyzed the outcome of a series of consecutive GSWB patients admitted alive to a level 1 trauma center in a safe high-income welfare country with a low rate of homicidal gun violence.

**Methods:**

Patients admitted due to a GSWB to the HUS Helsinki University Hospital during 2000–2012 were identified from hospital discharge registry and log books of the emergency room and ICU. CT scans and medical records of these patients were reviewed. Univariate analysis and backward logistic regression were performed, and their results compared with that of a systematic literature review of factors related to the outcome of GSWB patients.

**Results:**

Sixty-four patients admitted alive after GSWB were identified. Eighty percent had self-inflicted GSWB, 81% were contact shots, and 70% were caused by handguns. In-hospital mortality was 72%. Factors associated with mortality in our series were low GCS (≤ 8) at admission, transventricular bullet trajectory, and associated damage to deep brain structures, as reported before in the literature. Of the 64 patients admitted alive, 42% (27/64) were admitted to ICU, 34% (22/64) underwent surgery, and in 25% (16/64), craniotomy and hematoma evacuation was performed. Mortality in the surgically treated group was 32% but near 100% without surgery and ICU treatment. Median GOS in the surgically treated patients was 3 (range 1–5).

**Conclusions:**

GSWB caused by contact shot from handguns has a high mortality rate, but can be survived with reasonable outcome if limited to lobar injury without significant damage to deep brain structures or brain stem. In such GSWB patients, initial aggressive resuscitation, ICU admission, and surgery seem indicated.

**Electronic supplementary material:**

The online version of this article (10.1007/s00701-019-03952-y) contains supplementary material, which is available to authorized users.

## Introduction

Civilian brain injuries caused by gunshot wounds of the brain (GSWB) are rare (< 1/100,000 person years) in times of peace in most societies [9, 21,10]. Nevertheless, they do happen as the result of self-inflicted suicidal injuries, related to homicidal assaults, or as the result of accidents [21,10]. Globally, the number deaths attributed to firearm assaults is increasing [9,21,10]. Comparison of the incidence and etiology (suicidal, homicidal, accidental) of firearm-related injury between different countries shows great variation [9, 21,10]. Moreover, etiology of the GSW, as well as the type of weapon used, has been shown to greatly affect the outcome of the injury [13, 24].

The nature of being rare, but acute emergencies, makes it difficult to collect clinical experience on the management of GSWBs in a relatively safe high-income welfare state such as most European countries. Despite a large number of published observational GSWB patient series (supplemental Table S1), no established evidence-based guidelines for the management of these patients have been created. Several clinical and radiological factors that predict outcome and response to treatment have been identified in the prior literature (Table S1). However, as these studies originate from countries with varying etiologies of the GSWB, and some report the outcome of GSWB related to military conflicts (Table S1), it is not unequivocally clear how well the findings of prior literature can be applied to the management victims of civilian GSWB in high-income welfare countries in general considered safe.

Finland is a high-income welfare state [36] with a low rate of gun-related homicidal violence (1013) but by European standards a relatively high number of guns per inhabitants [34]. Most of these are hunting weapons or related to sport shooting due to the strict gun ownership policy [34]. As such, the etiology and nature of GSWs observed in Finland likely differs from that of conflict regions or less stable societies and likely reflect the situation in other similar high-income welfare states such as other Nordic and Central European countries. Prior epidemiological studies of GSWs in Finland have reported a 5.1/100,000 person year incidence for GSWs requiring hospitalization during 1985 to 1989 [3], with the incidence decreasing to 2.6/100,000 person years by 2003 [22]. Since 35% of these GSWs involved the head and neck, and 14% the brain [3, 22], the estimated incidence of GSW-related brain injury (GSWB) patients admitted to a hospital alive during these study periods would be 0.7 and 0.4/100,000 person years, respectively.

We studied the injuries and outcomes of a consecutive series of GSWB patients admitted alive to the largest level 1 trauma center in Finland, the Helsinki University Hospital, from 2000 to 2012. The aim of this study was to identify clinical factors predicting the survival of these patients and, through comparison with prior literature, to create an evidence-based algorithm for the management of GSWB patients encountered in neurosurgical units of high-income welfare states similar to Finland.

## Materials and methods

### Patients, data collection, and methods

In order to identify patients treated for GSWB at HUS Helsinki University Hospital (HUS), the hospital registry for discharge diagnoses was searched for gunshot injuries. Following this, the log books of the neurosurgical operating room and the emergency room were searched for patients treated for GSWB. The medical records and computerized tomography (CT) scans of identified patients were then reviewed. This study was approved by the Ethical Review Board of the Hospital District of Helsinki and Uusimaa that waived the need for informed consent.

Altogether, 64 patients with intracranial GSWB were treated at the Helsinki University Hospital during a 13-year period extending from January 2000 to December 2012 (Table 1). Only gunshot wounds caused by firearms (handgun, shotgun or rifle) were included in the study. The presence of an intracranial injury was confirmed by reviewing the CT scans performed in the emergency room (ER). In cases where no CT scan was performed, patients were included if an intracranial GSWB was clinically evident in the ER (entry wound visible).

**Table 1:** Type of injury and patient demographics

Variable	Self-inflicted (80%, 51/64)	Not self-inflicted/unkown (20%, 13/64)	*P* value
Age: median (min–max)	51 (18–86)	33 (20–74)	0.102
Gender (% males)	96.1% (49/51)	76.9% (10/13)	0.053
Weapon used			
Handgun	68.6% (35/51)	76.9% (10/13)	0.501
Shotgun	2.0% (1/51)	0% (0/13)	0.501
Rifle	11.8% (6/51)	0% (0/13)	0.501
Shot distance			
Contact	96.1% (49/51)	23.1% (3/13)	< 0.001
< 5-m distance	3.9% (2/51)	53.8% (7/13)	< 0.001
> 5-m distance	–	–	–
Outcome			
Death	77.6% (38/49)	61.5%(8/13)	0.291
GOS: median (min–max)	1 (1–5)	1 (1–5)	0.051

**Fig. 1:**
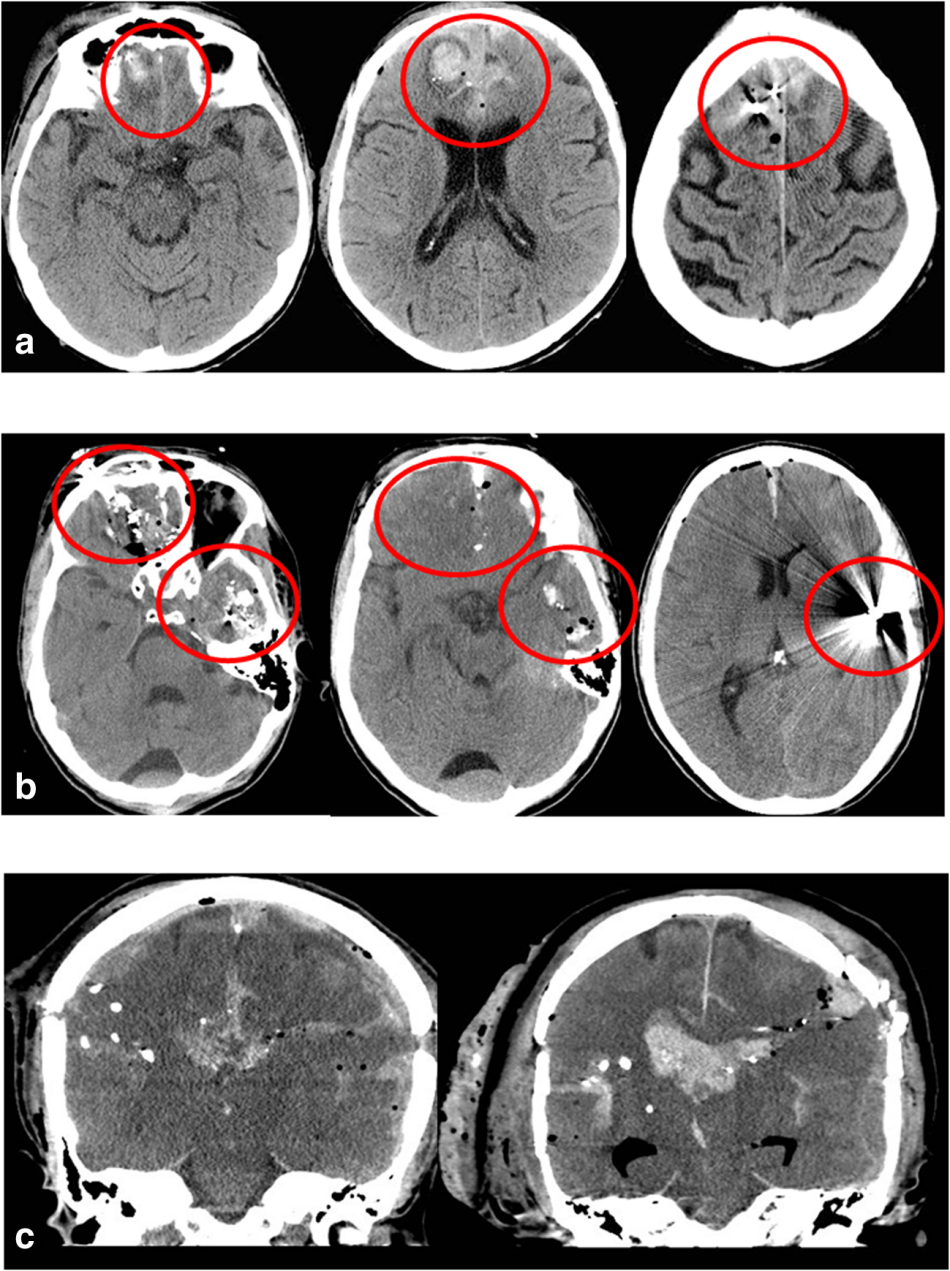
Examples of head CT scans in patients with gunshot wound with brain injury (GSWB). Patient A suffered injury of both frontal lobes without CT scan visible damage to the basal ganglia, the thalamus, or the ventricles. The three serial axial sections demonstrate the bullet path through the frontal lobes (shot through the palate, bullet remaining intracranially in the right superior frontal gyrus). Patient B demonstrates another example of GSWB causing damage to multiple lobes (both frontal lobes and the left temporal lobe) without affecting the basal ganglia, thalamus, or the ventricles. The injuries of Patient A and B are survivable despite extensive damage. The coronal CT scan sections in C demonstrate examples of the bullet tract passing through the ventricles in two different patients. This kind of gunshot injury was clearly associated with poor prognosis and can be deemed unsurvivable in most cases

The CT scans (Fig. 1) were retrospectively reviewed by a neurosurgeon (J.F.) and assessed for entry and exit sites, presence of extra-axial or intra-axial hematoma, edema, midline shift, and extent of injury scored on an ordinal scale as follows: (1) only lobar injury, (2) lobar injury and damage to the deep brain structures (ventricles, basal ganglia, ventricles), (3) damage to the midbrain and/or brain stem.

Data on neurological status (Glasgow Coma Scale [GCS] score), presence of extracranial injuries, and of blood counts on admission was collected from medical records (O.F.), as well as data describing the mechanism of injury and the type of weapon used. In addition, the medical records were reviewed for surgical procedures and outcome. The primary outcome variable was discharge alive from the hospital.

### Statistical analyses

Median and interquartile ranges (IQR) were calculated for continuous nonparametric variables and differences were compared with the Mann-Whitney *U* test. Categorical parameters are presented as numbers with percentages and groups compared using *χ*^2^ or Fisher’s exact tests, as appropriate. Logistic regression with backward selection was used for multivariate analysis. Backward selection instead of forward selection was used because the size of the series limited the number of possible variables included in the initial models. These variables were selected according to their significance in the univariate analysis. The statistical analyses were performed using IBM SPSS version 24 statistical software.

### Literature search

In order to identify relevant prior studies on the outcome of GSWB patients, we searched Pubmed with the following search terms: gsw, gunshot wound, gunshot, firearm, brain, intracranial, head. The titles and abstracts of the identified studies were reviewed, and original studies describing clinical series of GSWB patients included to Table S1 (supplemental material). In addition, we reviewed the reference lists of relevant review articles in order to complement the literature search.

## Results

### Type of injury and patient outcome

Of GSWBs in this study, 80% (51/64) were self-inflicted, 81% (52/64) were contact shots, and most were caused by handguns (45/64, 70%). Patient demographics are shown in Table 1. Our study included only patients that were admitted alive. Of them, 72% (46/64) died at the hospital. Of the 18/64 that were alive at discharge, 11 had sustained GSWBs from a handgun, two from a rifle, and none from a shotgun. For the remaining five, the type of gun was undetermined.

### Clinical presentation and patient outcome

The average time from the injury to hospital admission was 1.6 h (median 1.5 h, range 45 min–4 h 36 min). Clinical presentation on admission is summarized in Table 2. In this series, 20% (13/64) of patients were intubated in the field before admission. Of those that were not intubated in the field, 37 had a GCS score ≤ 8 on admission. GCS on site was significantly associated with GCS at admission (*p* < 0.001) and had decreased during transportation to the hospital in 25% (12/48) of those patients for whom both GCS on-site and at admission could be determined. Other life-threatening injuries were rare (thoracoabdominal 1/64). Blood loss as determined by hemoglobin (137 vs 114, *p* = 0.293 for GCS > 8 and GCS ≤ 8 respectively) or hematocrit (40 vs 34, *p* = 0.564 for GCS > 8 and GCS ≤ 8 respectively) at admission did not associate with neurological status.

**Table 2:** Clinical presentation and outcome. Patients are stratified according to their level of consciousness on admission into those with Glasgow Coma Scale (GCS) > 8 or ≤ 8, since GCS ≤ 8 signifies a decreased level of consciousness indicating intubation and mechanical ventilation to secure the airway and ensure sufficient ventilation, especially in patients with brain trauma. Intubation and mechanical ventilation require sedation, and in our series, 13 patients were intubated in the field before admission. For the remaining 51 patients, GCS score on admission could be determined for 48 patients. Of note is the observation that although subdural (SDH) or parenchymal hemorrhage (ICH) was observed in most patients, in most of them, the hematoma was not large enough to cause significant expansive effect indicated by midline shift (presence of midline shift indicates a > 1-mm midline shift). This was observed also in GCS ≤ 8 patients, and thus it can be concluded that although present in most patients, intracranial hematoma is usually not the cause of decreased level of consciousness in GSWB patients. In the GCS ≤ 8 patients in whom midline shift was present to measurable extent (*n* = 10), median shift was 9 mm (range 3–16 mm). Due to the retrospective nature of our study, we were not able to determine all the study variables accurately for all the patients. Because of this, the results are reported so that both the number of index cases and the number of patients from whom we were able to gather the information are given

Variable	Status at hospital admission		*p* value
	GCS > 8	GCS ≤ 8	
Documented on-site loss of consciousness	0.0% (0/9)	94.9% (37/39)	< 0.001
GCS on site: median (min–max)	15 (12–15), *n* = 9	3 (3–10), *n* = 39	< 0.001
GCS on admission: median (min–max)	15 (11–15), *n* = 9	3 (3–7), *n* = 39	< 0.001
Thoracoabdominal injuries	0% (0/9)	2.6% (1/39)	< 0.001
Hypotension (during transport)	14.3% (1/7)	35.5% (11/31)	0.395
Edema	33.3% (3/9)	93.8% (30/32)	< 0.001
Presence of any midline shift	33.3% (3/9)	40.6% (13/32)	1.000
SDH	55.6% (5/9)	68.8% (22/32)	0.692
ICH	55.6% (5/9)	87.5% (28/32)	0.054
Wound tract through the ventricles in CT	0.0% (0/9)	61.5% (24/39)	< 0.001
Wound tract through the basal ganglia in CT	22.2% (2/9)	71.9% (23/32)	0.023
Wound tract through the thalamus in CT	0.0% (0/9)	43.8% (14/32)	0.049
Wound tract in CT through the brain stem/medulla oblongata	0.0% (0/5)	23.1% (3/13)	0.383
Surgical intervention	77.8% (7/9)	20.5% (8/39)	0.002
GOS: median (min–max)	5 (3–5), *n* = 6	1 (1–5), *n* = 38	< 0.001
Death	0,0% (0/9)	92.3% (36/38)	< 0.001

Death during hospitalization was significantly associated with GCS ≤ 8 on admission (*p* < 0.001), and of those with a GCS > 8 registered on admission, none died (0/9) (Table 3). Over 90% of those with a GCS ≤ 8 on admission had edema in CT scans compared to only 3/9 of those with GCS > 8. Expansive hematoma or edema causing midline shift did not explain the lower GCS score on those with GCS ≤ 8 on admission but a wound tract passing through the ventricles (*p* < 0.001) or the thalamus (*p* = 0.049) was associated with GCS ≤ 8.

**Table 3:** Association of clinical presentation with the radiological presentation and outcome. Of the 64 patients admitted alive with GSWB, 55 underwent a head CT scan. For the remaining 9 patients, prognosis was deemed so poor based on the clinical status and examination that only palliative treatment was administered after admission and no head CT scan was performed. The extent of injury visible in the head CT scans was stratified according to whether only lobar injury was observed, or whether deeper brain structure were affected or the mesencephalon and brain stem were affected as well. The affected lobes, presence of parenchymal (ICH) or subdural (SDH) hematoma, as well as the presence of edema were assessed from the CT scan. In addition, the presence of any midline shift was scored (yes or no) as an indicator of the expansive nature of concomitant hematoma or edema. Somewhat surprisingly most patients did not present with midline shift despite most of them presenting with hematoma or edema, suggesting that most of the hematomas were not very expansive. Due to the retrospective nature of our study, we were not able to determine all the study variables accurately for all the patients. Because of this, the results are reported so that both the number of index cases and the number of patients from whom we were able to gather the information are given

Variable	Extent of injury			*p* value
	Lobar	Through ventricles or basal ganglia or thalamus	Midbrain and/or brain stem affected	
GCS on admission: median (min–max)	12 (3–15), *n* = 13	3 (3–15), *n* = 25	3 (3–6), *n* = 3	0.014
GCS ≤ 8	40.0% (6/15)	69.7% (23/33)	100% (3/3)	0.010
Multiple lobes affected	88.2% (15/17)	94.3% (33/35)	100% (3/3)	0.674
Frontal	88.2% (15/17)	88.6% (31/35)	100% (3/3)	1.000
Parietal	29.4% (5/17)	34.3% (12/35)	66.7% (2/3)	0.523
Occipital	5.9% (1/17)	5.7% (2/35)	0% (0/3)	1.000
Temporal	76.5% (13/17)	71.4% (25/35)	100% (3/3)	0.884
Cerebellar	11.8% (2/17)	0% (0/35)	33.3% (1/3)	0.022
Bullet exit wound	33.3% (5/15)	58.6% (17/29)	33.3% (1/3)	0.324
Secondary missiles	93.8% (15/16)	97.1% (34/35)	100% (3/3)	0.584
ICH	41.2% (7/17)	97.1% (34/35)	66.7% (2/3)	< 0.001
SDH	47.1% (8/17)	65.7% (23/35)	33.3% (1/3)	0.304
Edema	41.2% (7/17)	85.7% (30/35)	100% (3/3)	0.003
Presence of any midline shift	29.4% (5/17)	45.7% (16/35)	33.3% (1/3)	0.588
Surgical intervention	64.7% (11/17)	28.6% (10/35)	33.3% (1/3)	0.004
GOS: median (min–max)	3 (1–5), *n* = 13	1 (1–5), *n* = 33	1 (1–1), *n* = 2	0.003
Death	37.5% (6/16)	84.8% (28/33)	66.7% (2/3)	0.001

### Radiological presentation and patient outcome

The radiological presentation of the GSWB in relation to status at admission, extent of injury, or outcome of the patient, are shown in Table 3. Of patients included in this study, 86% (55/64) underwent a head CT scan. In the case of the remaining 9 patients, no CT scan was performed because the prognosis of the injury was deemed so poor based on clinical status (GCS 3) and examination (entry and or exit wound) that the admitting physician withdrew from further intensive care.

Among those, GSWB had damaged multiple lobes in 93% (51/55). In 29% (16/55), the injury was only lobar, whereas in the remaining 71% (39/55), deep structures such as the basal ganglia, the thalamus, or the ventricles were affected as well. Of patients with deep brain structure damage, 100% were either intubated and sedated or had a GCS ≤ 8 at admission. Damage to deep structures, especially the ventricles, was also clearly associated with higher mortality during hospitalization (*p* = 0.001).

### Effect of interventions on outcome

Of the 64 patients admitted with vital signs, 42% (28/64) were admitted to ICU. The ICU care consisted of monitoring the level of consciousness (LOC), intubation and controlled ventilation if LOC decreased (GCS ≤ 8), control of systemic blood pressure to ensure cerebral perfusion pressure over 60 mmHg, and hyperosmolar therapy with hypertonic saline or mannitol, complemented by surgery (hematoma evacuation, wound debridement, ventriculostomy, or ICP monitoring as needed) in 22 patients. Craniotomy and hematoma evacuation with possible revision of the contused brain parenchyma was performed in 16 patients (Table 4). The reason from withdrawing from intensive care or surgical procedures was poor clinical status and extensive injury diagnosed on admission (GCS 3 or 4), with the exception of 1 patient in whom bullet had not penetrated the skull. In this patient, GCS was 15 on admission, despite contusive changes observed in the CT. Not surprisingly, mortality was high in those patients that did not receive surgical interventions (Table 4), and would have reached 100% if the patient mentioned above was excluded.

**Table 4:** Treatment and overall outcome. Of the 64 patients admitted alive with GSWB, 27 were admitted to the ICU and 22 underwent surgery (ICP monitor placement, ventriculostomy, craniotomy and hematoma evacuation, wound debridement). Those patients who underwent surgery had a significantly better level of consciousness on admission, as determined by the Glasgow Coma Scale (GCS). GCS could not be accurately retrospectively determined from those patients that were intubated and sedated in the field prior to hospital admission, and therefore, GCS on admission is given for 48 patients. Those 37 patients not admitted to the ICU were deemed unsalvageable based on clinical status and examination and 9 of these patients did not undergo a head CT scan. Thus, the course of the wound tract could be evaluated in the CT scans of 55 patients. Moreover, data on the clinical outcome could not be determined retrospectively from the patient records of all patients, and thus, outcome data is presented for only 49 patients. 1/22 of the surgically treated patients underwent only placement of an ICP monitoring probe, and 1/22 underwent only reconstruction of the anterior skull base. Superficial wound revision only (*) was performed for 3 patients admitted with GCS 15. In 2 of these patients, the bullet remained in the bone, but there was a contusion hemorrhage in the adjacent temporal lobe that did not require operative treatment. The 1 patient that did not die despite not undergoing surgery (**) had right-sided frontotemporal contusions that were treated conservatively, and in this case, the bullet had not penetrated the skull bone

Variable	Treatment intensity		*p* value
	Surgery (*n* = 22)	No surgery (*n* = 42)	
GCS on admission: median (min–max)	7 (3–15) *n* = 15	3 (3–15) *n* = 33	< 0.001
Wound tract through the ventricles, the basal ganglia, or the thalamus in CT	40.9% (9/22)	72.7% (24/33)	0.026
Craniotomy and evacuation of hematoma or contusion and hematoma	16/22	–	NA
Bullet removal	11/22	–	NA
Superficial wound revision*	3/22	–	–
Death	32% (7/22)	98% (39/40)**	< 0.001
GOS: median (min–max)	3 (1–5), *n* = 17	1 (1–5), *n* = 40	< 0.001
Return to prior occupation	27% (3/11)	0% (0/38)	0.002

Of the surgically treated patients, 46% (10/22) had only lobar injury. In 17 patients with only lobar injury, there was a statistically nonsignificant trend for better outcome among those who were surgically treated (80% discharged alive in the surgically treated group vs 33% in the conservative group, *p* = 0.118). In 38 patients with injury to deep brain structures, surgery was associated with better outcome (58% discharged alive in the surgically treated group vs 4% in the conservative group, *p* < 0.001).

### Multivariate modeling

In a logistic regression model including age, GCS at admission as a continuous variable, and extent of injury in CT scan (ordinal scale according to Table 3), only GCS at admission was significantly associated with survival (OR 1.3, 95% CI 1.1–1.4, *p* < 0.001)*.* In a model including age, GCS at admission as a continuous variable, and extent of injury categorized according to the wound tract passing through a single or multiple lobes, the basal ganglia or the thalamus, or through the ventricles, only GCS at admission remained significantly associated with survival after backward selection (OR 2.7, 95% CI: 1.1–7.0, *p* = 0.037). However, in a model including the same variables but with GCS at admission as a binary variable (GCS ≤ 8 or > 8), only wound tract passing through the ventricles remained significantly associated with death after backward selection (OR 38.0, 95% CI 1.7–870.5, *p* = 0.023). When surgery was included in the logistic regression model, neither GCS at admission nor wound tract passing through the ventricles remained significant, although all the three variables were selected as the remaining three variables in backward selection.

## Discussion

We describe the outcome of patients suffering from civilian gunshot wounds of the brain, admitted as a consecutive non-selected clinical series to a single tertiary trauma center with a catchment area of approximately 2 million in a high-income welfare state with a low overall incidence of homicidal firearm violence [21,10]. In our series, most patients were males that had a self-inflicted GSWB caused by a contact gunshot from a handgun. In general, handguns fire lower velocity bullets and lighter bullets than rifles intended for hunting or military purposes, and thus also cause lower energy GSW than rifles [29].

Despite the relatively large catchment population of our institution (2 million), only 64 patients were admitted alive after a GSWB during the 13-year data collection period (2000–2012), leading to an estimated yearly incidence of 0.3 cases/100,000 patient years. Two prior epidemiological studies of GSWB in Finland report an incidence of 0.7 and 0.4 GSWB patients admitted alive/100,000 patient years during the 1980s and the 1990s respectively [3, 22]. Comparison of these prior studies with ours suggests a decreasing trend in the incidence GSWB patients in Finland during the past three decades. This interpretation is in line with a prior study performed at our hospital in the 1970s, which reported 90 GSWB patients admitted alive during an 8-year time period despite a smaller catchment area population [12].

### Comparison of the GSWBs admitted alive in our series with previously reported fatal GSWs from the same catchment area

A prior forensic pathology study of GSW victims in Southern Finland, the catchment area of HUS, reported 348 fatal GSWs during a 7-year period from 1995 to 2001, or approx. 49–50 fatal GSWs per year [28]. Of these fatal GSWs, 88% (306/348) were in the head [28]. Extrapolating from this prior study, 637–650 fatal GSWs of which 561–572 are GSWBs, would have been expected during our study period in our catchment area. Comparison of this estimate with our 64 GSWB patients admitted alive suggests a prehospital mortality of 89% for GSWBs in our catchment area during our study period.

Of the fatal GSWs previously reported from our catchment area, 88% (305/348) were suicides [28]. In 92% of these suicides, the GSW was in the head and entry wound most often in the mouth. In our series, 80% of the GSWBs were self-inflicted, which suggests that the etiology of these two series does not significantly differ. In both series, most GSWs were self-inflicted with handguns (28 and Table 1). Of interest is the observation that in the prior series of fatal GSWs from our catchment area, 15% had received medical treatment to their GSW prior to their death [28]. Of the victims of fatal handgun GSWs, 18% had received medical treatment prior to death, but only 3% of the shotgun and 2% of the rifle GSW victims [28]. Together, these series demonstrate that in spite of the high prehospital case fatality rate, a non-negligible proportion of especially handgun-inflicted GSWBs will be admitted alive to a hospital—and thus will require careful assessment of the prognosis and intensity of treatment by the admitting physician.

### Neurological status (GCS) at admission as a prognostic indicator for survival

In our series of patients that survived to the hospital, GCS on admission was a strong predictor of outcome. Due to the limited statistical power of our series, and the comparatively large number of variables possibly affecting the survival in statistical analysis, we categorized patients according to whether their GCS on admission was less or equal to, or above 8. This categorization is clinically very relevant, since in patients with traumatic brain injury GCS 8 or below is generally considered as a decreased level of consciousness requiring endotracheal intubation [2] and mechanical ventilation, which in turn indicates sedation and thus impairs the continuous monitoring of GCS. In practical terms, GCS 8 or above corresponds to a patient that after stimulation and arousal obeys commands.

GCS on admission was identified as a significant predictor of outcome in 31 of the studies found in our literature search (Table S1), including the only meta-analysis on the predictors of GSWB outcome [20]. Low GCS may indicate hematoma- or edema-induced compression of diencephalic and brain stem structures maintaining consciousness, or direct damage to these structures by the wound tract. In our series, low GCS (≤ 8) on admission was associated with penetrating injury to the deep structures or ventricles, rather than with expansive hematomas compressing the brain. Multivariate analysis with backward logistic regression suggested that damage to the deep structures or transventricular wound tract explains the association of low GCS with mortality in our series.

### Radiological markers of poor prognosis—intraventricular wound tract

In addition to our series, transventricular wound tract was associated with poor outcome in 14 of the prior studies identified in our literature search (Table 1), including the meta-analysis. The high energy of a bullet penetrating the tissues can produce a shockwave spreading in the tissues and causing neurological injury even when the actual wound tract is remote from the central nervous system [27]. Because the brain ventricles are filled with cerebrospinal fluid that surrounds all parts of the brain and is largely water which is an excellent conductor of shockwaves [30], the energy of a bullet passing through the ventricles might be transmitted more widespread in the brain tissue than the energy of a bullet passing only through brain tissue. Neural damage caused by the spread of shockwaves via the venous system from an extracranial blast exposure has been demonstrated in a controlled experimental setting [32], demonstrating the concept of neuronal injury caused by shockwaves spreading to the brain through body fluids.

Another important factor explaining the association of transventricular bullet tract with poor outcome is the proximity of the ventricles to the diencephalon (thalamus, hypothalamus, subthalamus) and the fact that the high kinetic energy released by a bullet passing through tissues is transmitted beyond the wound tract. In most tissues, this energy released by the bullet creates through cavitation a temporary cavity that despite its name reflects an area of permanent tissue damage. Inside the skull, however, the confined nature of the cranium minimizes this cavitation [14]. Instead, the energy released from the bullet is diverted into shearing forces that injure the tissue as much as cavitation and extend the tissue damage to structures beyond the original wound tract [14]. Thus, a transventricular bullet tract will likely cause also diencephalic damage even if the diencephalon is not directly penetrated.

### Implications for the management of gunshot wounds of the brain

Whether aggressive emergency room management, surgical treatment of GSWB, or ICU admission is indicated or not, and in which cases it is, is a challenge to the physician facing these patients at the ER. Bullet tract penetrating the brain stem, the midbrain, or both thalami (diencephalon) can be considered unsurvivable due to destruction of life-preserving structures. However, GSWB causing only lobar injury or limited degree of injury to deeper brain structures is survivable (Table 3 and refs. 6, 11, 17, 26), and therefore, admission to intensive care surgical treatment should be considered. Although low GCS (≤ 8) is associated with poor outcome both in our prior literature and in our series, it is worth noting that in a multivariate model including transventricular bullet tract, low GCS on admission lost its significance. This was observed also in a multivariate model including surgical intervention. These results suggest that GSWB should not be determined unsurvivable just based on low GCS, but only after a CT scan demonstrates extensive damage to vital brain structure. This in turn implies that GSWB patients should be treated as other traumatic brain injury patients until a CT scan confirms unsurvivable brain damage, which means that GSWB with low GCS (≤ 8) should be intubated and ventilated to avoid consequences of hypoventilation and subsequent raise in ICP [2, 8].

GSW to the head may cause three types of brain injury: (1) a closed head injury related to the blunt force trauma caused by impact of the bullet, (2) penetrating brain injury caused by laceration of brain tissue by the bullet and the shearing forces resulting from the kinetic energy of it, and (3) perforating head injury in which the bullet causing the penetrating brain injury exits the skull and thus does not release all of its energy inside the cranium. Management of these brain injuries should follow the guidelines for the management of severe traumatic brain injury by the Brain Trauma Foundation [2,4,25], including removal of expansive hematoma, control of intracranial pressure (ICP), and maintenance of cerebral perfusion pressure > 60 mmHg to ensure sufficient cerebral blood flow and tissue oxygenation. Adherence to these guidelines improves survival in patients with traumatic brain injury [31].

**Fig. 2:**
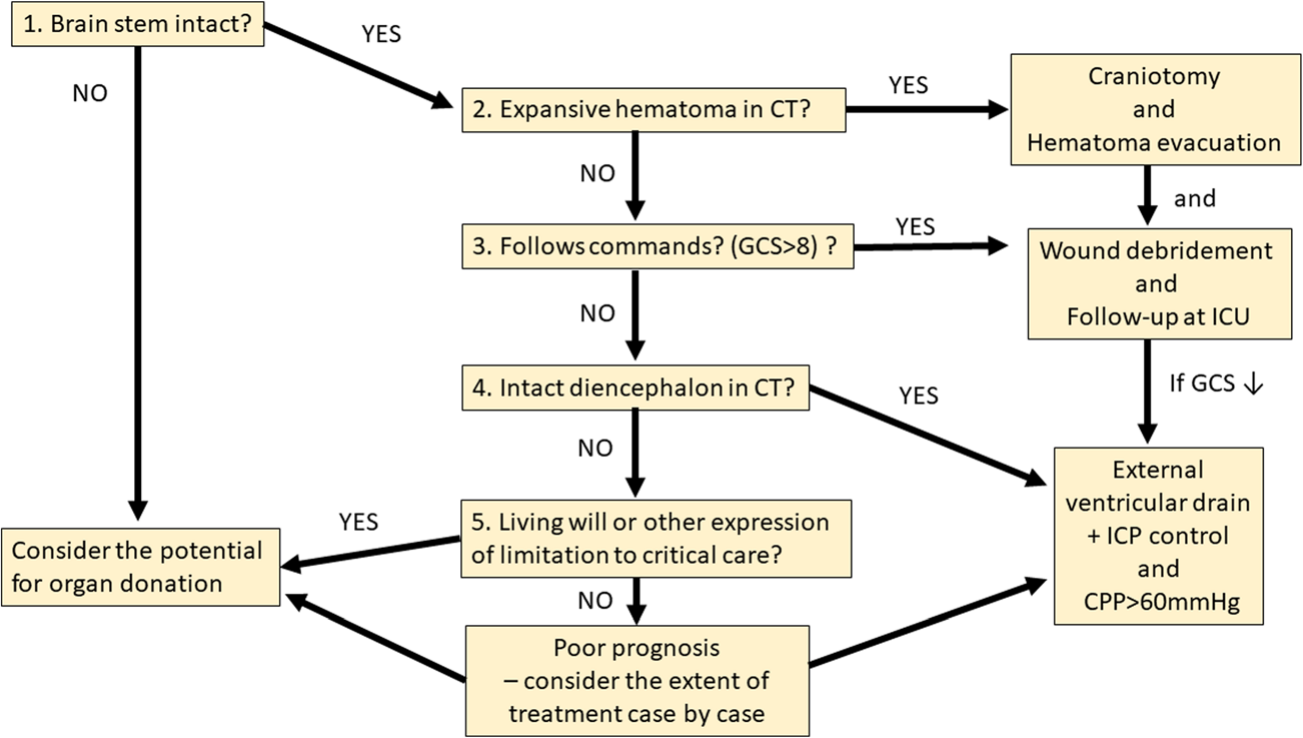
Proposed algorithm for the management of gunshot shot wounds with brain injury (GSWB). The most critical brain structure for survival are the brain stem and the midbrain. Whether they are intact or not can be clinically assessed despite sedation (question 1). When the brain stem and midbrain are intact after GSWB, control of intracranial pressure (ICP) is paramount and as the first step expansive extra-axial or intraparenchymal hematomas should be removed (question 2). If the patient has a decreased level of conciousness (GCS ≤ 8) in spite of no expansive hematoma (question 3.) one needs to look at the CT scan for signs of transventricular bullet tract (e.g., intraventricular hemorrhage) or diencephalic damage (bullet tract passing through the thalamus, the hypothalamus, or the subthalamus, or contusions in these structures, question 4) that are indicators of unsurvivable brain damage despite an intact brain stem. If there is no expansive hematoma to remove surgically, the level of consciousness is nevertheless GCS ≤ 8, and there is no sign of unsurvivable brain damage, and external ventricular drain (EVD) should be placed to monitor ICP and cerebral perfusion pressure (CPP) and treat both according to the Brain Trauma Foundation Guidelines [20]. Finally, in the case of intraventricular bullet tract or diencephalic damage apparent in the CT and suggesting unsurvivable injury, placement of and EVD might be considered, since in our series, some patients survived despite such injuries. However, in this situation, the unlikely survival is likely to result in serious neurological deficits, and thus, overaggressive management is to be avoided so as not to prolong suffering against the patient’s own will [34]. Therefore, it should be considered whether the patient had a so-called “living will” or by other means had expressed his/her will regarding limitation or withdrawal of care in the event of seriously incapacitating injury (question 5.). Whether suicide attempt as the etiology of injury can be considered as such an expression of will is to be discussed

In an autopsy series of 18 patients that died due to acute penetrating brain injury, Lillard et al. showed that all of them had undergone brain herniation [19]. This implies that control of ICP is critical for patients with penetrating brain injury. Later Kirkpatrick and DiMaio showed signs of high ICP in postmortem examination 93% (39/42) of patients that died after GSWB [16]. Observational ICP monitoring data has shown an increase in ICP following GSWB (reviewed in [20], and that successful treatment of ICP is associated with survival [31, 20, 23, 25]. Together, these studies suggest that intensive care with medical and surgical measures to control ICP is indicated in GSWB victims when the injury cannot be undisputably considered unsurvivable due to destruction of critical structures (Figs. 1 and 2). Our data supports this concept, since we observed a clear trend for better outcome in GSWB patients with only lobar injury who were surgically treated, as well as a statistically significant difference in survival among those GSWB patients who had concomitant injury to deeper brain structures but nevertheless underwent surgery. A proposed algorithm for the management of GSWB patients is presented in Fig. 2.

### Lessons learned from the combat field

DuBose et al. reported the outcomes of patients who suffered traumatic brain injury by various mechanisms during a relatively recent modern era military conflict, including 118 patients with GSWB [7]. Of these, 35% underwent some form of surgical treatment and the overall mortality was remarkably low, only 7% [7]. Overall, the mortality of penetrating brain injury in this series was close to tenfold lower than among matched civilian injury [7]. Although the presence of helmets or other protective gear absorbing part of the bullet energy might explain in part some of this very significant difference, it is clear that in the series described by DuBose et al., ICP monitoring, skull debridement, decompressive craniectomy, and overall neurosurgical intervention was performed much more frequently than in the control group of civilian injuries [7]. This strongly suggests that aggressive surgical management of GSWBs significantly improves outcome. Another probably significant factor contributing to the better survival of the military GSWBs described by DuBose et al. is the early attention to ICP management and mechanical ventilation [1] and subsequently better implementation of the Brain Trauma Foundation guidelines [2] than in some series of civilian injuries, including ours.

### Comparison of civilian gunshot wounds of the brain with similar brain injury of different etiology

According to Valadka et al., who compared the outcome of GSWB with similar brain injuries caused by other mechanisms, salvageable GSWB patients can make a similar recovery to brain injury patients with similar injuries from other mechanisms [35]. However, GSWB leads to higher ICP and lower CPP than other mechanisms of brain injury [35], which suggests that salvageable GSWB patients would especially need neurointensive monitoring and care. Considering that the reported mortality rates of GSWB vary from < 10% to > 90% depending on the patients and injuries included in the studies [1, 6, 7, 11, 15, 16, 17, 18, 19, 20, 23, 24, 26, 33, 35], ICU admission of GSWB patients seems indicated since some of these patients survive their injuries. Moreover, in case the GSWB leads to brain death despite therapeutic efforts, many of these patients are potential organ donors and thus admission to the intensive care unit and ventilator treatment is justified even if the prognosis seems poor [5].

However, also the high likelihood for mortality of GSWB and the significant cost of intensive care needs to be considered, and unnecessary prolongation of ICU treatment should be avoided when no sign of neurological recovery is seen. In the USA, gunshot violence is among the most expensive causes of hospitalization [18,15].

It is worth noting that in a prior clinical series of GSWB patients treated at our hospital in the pre-CT era when neuroradiological examination was based on angiography and neurointensive care was less developed (in the 1970s), the level of consciousness on admission was predictive of the outcome and being alert on admission was associated with meaningful recovery [12]. In addition to the level of consciousness on admission, in this older series, bullet tract crossing the midline in coronal or sagittal plane was associated with poor outcome [12]. This corresponds to multilobar injury or transventricular bullet tract associated with poor outcome in more modern series (Table S1) including ours. Comparison of our current data with this historical series from the same institution and catchment area supports the intuitive conclusion that extent of the injury, visible both in the clinical status as well as in the radiological examinations, will remain the main factor determining the outcome of GSWB regardless of advancements in neurointensive care. However, our conclusion has changed from “prevention is the only worthwhile approach” in the 1970s to ICU admission and surgical treatment are justified despite poor prognosis because of the possibility of meaningful recovery depending on the extent of the brain trauma. Furthermore, the increasing possibility of organ transplantation justifies the ICU admission even if initial prognosis seems poor.

## Conclusion

Although GSWBs are rare in stable high-income welfare states such as most European countries, emergency room physicians and on-call neurosurgeons will be faced with them. Both our series as well as many of the previously published series demonstrate that GSWB without damage to deep brain structures can be survived with reasonable outcome. Decreased level of consciousness as defined by GCS ≤ 8 and wound tract extending to the ventricles were associated with death in GSWB patients admitted alive to our hospital, as well as in several previously published series. Our data, however, also shows that GSWB can in some cases be survived despite GCS ≤ 8 on admission or wound tract extending to the ventricles, so initial aggressive medical and surgical management of ICP may be indicated even with low level of consciousness on admission, and in selected cases even with the bullet tract penetrating the deep brain structures or the brain ventricles. To facilitate the decisions that need to be made in the emergency room on admission of GSWB patients, as well as to guide the overall clinical management of these patients, we present a treatment algorithm based on the outcome of GSWB patients in our series as well as systematically reviewed prior literature.

## Electronic supplementary material

ESM 1 (DOCX 39 kb)
