# Supplementary material for: Outcome and rational management of civilian gunshot injuries to the brain—retrospective analysis of patients treated at the Helsinki University Hospital from 2000 to 2012
Source: Acta Neurochir (Wien). 2019 May 25;161(7):1285–95. doi: 10.1007/s00701-019-03952-y (PMC6581925; doi:10.1007/s00701-019-03952-y)
Supplement: Supplementary file 1 — (DOCX 39 kb) [file 701_2019_3952_MOESM1_ESM.docx]

Supplemental Table 1.

A literature search using keywords gsw, gunshot wound, gunshot, firearm, brain, intracranial head was done. The search was limited to publications published after 1990, with the exception of accepting a previous publication about gunshot wounds of the brain from Finland by Hernesniemi J.

| **First author and PMID** | **Nro of patients** | **Setting** | **Time Period** | **Factors predicting outcome identified in the study** |
| --- | --- | --- | --- | --- |
| Hernesniemi J  517178 | 90 | Civilian | 1968-1977 | 1. Level of consciousness on admission 2. Wound tract through mid-coronal, mid-sagittal or both planes |
| Stone JL  8584151 | 480 | Civilian | 1983-1992 | 1. Even patients with severe neurological deficits and massive cerebral damage can benefit from aggressive treatment and make satisfactory recoveries. |
| Kim TW  17563664 | 13 | Civilian | 1983-2005 | 1. GCS < 8 2. Correlation between the presence of tranventricular or bihemispheric trajectory and poor outcome |
| Shaffrey ME  1588617 | 62 | Civilian | 1984-1990 | 1. Admission GCS 2. Initial pupillary response 3. Abnormal coagulation states 4. Neuroradiologic examination |
| Nathoo N  12480225 | 26 | Civilian | 1986-2000 | Good outcome:   1. Primary missile entry of the infratentorial compartment   Bad outcome:   1. Supratentorial to infratentorial missile trajectory |
| Helling TS  1548730 | 89 | Civilian | 1987-1989 | 1. No operation fared worse |
| Tsuei YS  15813246 | 16 | Civilian | 1988-2002 | 1. GCS score on admission 2. Extent of brain injury as visualized by CT scan |
| Jacobs DG  7618800 | 57 | Civilian | 1990-1992 | 1. GCS < 4 2. Respiratory rate < 10 3. Self-inflicted wounds 4. No differences clinically or demographically between non-surviving donors and non-donors |
| Gressot LV  24995781 | 119 | Civilian | 1990-2008 | 1. Age over 35 2. Nonreactive pupils 3. Bullet trajectory of bihemispheric (excluding bifrontal) 4. Posterior fossa involvement 5. ICP > 20 mmHg 6. GCS 3 or 4 at presentation |
| Strojnik T  15506305 | 37 | Civilian | 1992-2002 | 1. Post resuscitation GCS 3-8 2. Fixed and dilated pupils 3. Hypotension 4. Apnea 5. Bihemispheric, transventricular or multilobar lesion |
| Murano T  16447469 | 298 | Civilian | 1992-2003 | 1. Respiratory arrest on admission 2. Hypotension on admission 3. Transhemispheric and transventricular GSW. |
| Solmaz I  19621284 | 442 | Unknown | 1992-2008 | 1. Low GCS scores 2. Ventricular injuries 3. Bihemispheric injuries |
| Hofbauer M  20173654 | 85 | Civilian | 1992-2008 | 1. GCS score at admission 2. Pupil status/reaction 3. Hemodynamic situation 4. Respiratory situation 5. CT: Bi- or multilobar injury + intraventricular hemorrhage 6. Shot distance |
| Crandon IW  15622679 | 30 | Civilian | 1993-1998 | 1. GCS |
| Petridis AK  20309801 | 30 | Civilian | 1993-2008 | 1. Low GCS of 3-8 2. Fixed pupils 3. >2 bone fragments 4. Bilobar or posterior fossa/brainstem lesions 5. ICP > 45 mmHg |
| Döşoğlu M  10567959 | 47 | Civilian | 1994-1998 | 1. Hypovolemia 2. Respiratory depression 3. Bilateral pupil dilatation 4. GCS score 3-5 5. Central bihemispheric or transventricular injury 6. Multilobar injury 7. ICH, SDH, self-inflicted? (Vain abstrakti aukeaa) |
| Martins RS  12900108 | 319 | Civilian | 1994-2000 | Clinical:   1. Low GCS 2. Unilateral dilated pupil 3. Medium fixed pupils   CT:   1. transventricular or bihemispheric central type trajectory 2. Bilobar or multilobar injury |
| DeCuypere M  26728100 | 71 | Civilian | 1996-2013 | 1. Bilateral fixed pupils 2. Deep nuclear injury 3. Transventricular projectile trajectory 4. Bihemispheric injury 5. Injury to ≥ 3 lobes 6. Systolic blood pressure < 100 mm Hg 7. Anemia (hematocrit < 30%) 8. Glasgow Coma Scale score ≤ 5 9. Blood base deficit < -5 mEq/L |
| Khan MB  23313238 | 51 | Civilian | 1998-2011 | 1. Admission GCS 2. Nubmer of lobes involved |
| Stoffel M  20078527 | 214 | Civilian | 2000 October - 2005 May | 1. Loss of consciousness 2. Systolic BP < 100 mmHg or >= 200 mmHg 3. Brain spilling out of the wound (oozers vs non-oozers) |
| Livingston DH  24368351 | 6322 | Civilian | 2000-2011 | - |
| Maragkos GA  29877141 | 1774 | Civilian | 2000-2017 | Meta-analysis   1. Age > 40 years 2. Suicide attempt 3. GCS < 9 4. Bilateral fixed and dilated pupils 5. Dural penetration 6. Bihemispheric, multilobar or transventricular injury 7. ICP > 20 mmHg |
| Kim KA  16239886 | 37 | Civilian | 2001 June - 2002 December | 1. GCS score 2. Pupil irregularity 3. Bullet passage through a particular supra-dorsum sellar transventricular zone (4 cm above the dorsum sella) |
| Ambrosi PB  22415660 | 110 | Civilian | 2002-2005 | 1. Age over 40 years 2. Presence of unilateral pupil dilatation 3. Low GCS on admission 4. Presence of intracranial haematoma 5. Respiratory infection |
| Smith JE  24398079 | 813 (188 GSW) | Miltary | 2003-2011 | 1. GSWs are worse than blast injuries |
| Deng H  29855212 | 8148 | Civilian | 2003-2012 | 1. GCS 2. ISS 3. Hypotension 4. Firearm type 5. Injury intent 6. US geographic location |
| Onyia EE  28584512 | 52 | Civilian | 2004-2014 | 1. Postresuscitation Glasgow coma scale (GCS) score ≤8 2. Diencephalic, transventricular, and posterior fossa involvement |
| Bodanapally UK  25361486 | 55 | Civilian | 2005-2012 | Risk factors for an intracranial arterial injury on CT:   1. Entry wound over the frontobasal-temporal regions 2. Bihemispheric wound trajectory 3. Wound trajectory in proximity to the circle of Willis (COW) 4. Subarachnoid hemorrhage (SAH) 5. A higher SAH score 6. Intraventricular hemorrhage (IVH) 7. Higher IVH score |
| Joseph B  24055384 | 132 | Civilian | 2007-2011 | Better outcome:  1. Aggressive management with blood products (p = 0.02) and hyperosmolar therapy (p = 0.01) was independently associated with survival |
| Lin DJ  23061014 | 4 | Civilian | 2007-2011 | Good outcome predictors in patients with GCS < 5 on admission:   1. Time from injury to surgery < 1 h 2. Injury to noneloquent brain 3. Absence of injury to midbrain, brainstem and main vessels |
| Crutcher CL 2nd  27294759 | 111 | Civilian | 2008 January - 2013 October | Neither race nor intent (assault vs self-inflicted) predicted survival outcome |
| Martinez-Bustamante D  25986984 | 52 | Civilian | 2009 January - 2013 January | 1. GCS 2. State of the pupils |
| Kong VY  28612168 | 102 | Civilian | 2010-2014 | 1. Rural < urban patients, because: 2. Longer transport time & lower GCS on admission |
| Kong VY  28900668 | 102 | Civilian | 2010-2014 | Baragwanath mortality prediction score: ABC  A: admission blood pressure  B: brain matter spillage  C: consciousness level |
| Parrado Sánchez  28585791 | 32 | Civilian | 2011-2015 | 1. Low GCS on admission 2. Pupillary exam and light reflexes 3. ISS 4. Marshal score 5. On CT: absence of basal cisterns, presence of SAH 6. APACHE II |
| Can Ç  27903839 | 104 | Military | Unknown | 1. Midline shift 2. Pneumocephalus 3. Penetrating head injury 4. GCS =< 6 5. Intubation in the prehospital period |
| Valadka AB  10697090 | 71 + 541 | Civilian | Unknown | 1. GSWH similar global/metabolic pattern of injury as non-GSWH |
| Benzel EC  1870690 | 120 | Unknown | Unknown | 1. Level of consciousness on admission |
| Aarabi B  24506239 | 786 | Civilian | Unknown | 1. Admission GCS score 2. Trajectory of the missile track 3. Abnormal pupillary reaction to light 4. Patency of basal cisterns 5. Age 6. Intraventricular bleed |
| Aldrich EF  1298106 | 151 | Civilian | Unknown | 1. Intracranial hypertension 2. Midline shift 3. Compression or obliteration of the mesencephalic cisterns 4. Presence of subarachnoid blood 5. Intraventricular hemorrhage 6. Presence of hyperdense or mixed-density lesions greater than 15 mL, either bilateral or unilateral |
| Grahm TW  2259398 | 100 | Civilian | Unknown | 1. GCS after resuscitation |
| Kennedy F  8331716 | 192 | Civilian | Unknown | 1. GCS on admission |
| Rosenfeld JV  25446474 |  | Civilian & Military | Unknown | CLINICAL   1. GCS < 5 (post-resuscitation) on admission 2. Dilated, unreactive pupil(s) 3. Occipital entry wound 4. Brainstem injury 5. Injury to ‘eloquent’ brain 6. High-velocity missile injury (e.g., semiautomatic military-type weapons) 7. Hypotension on admission 8. Major intracranial vascular injury 9. High ICP 10. Onset of diabetes insipidus 11. Suicide attempt (because of close range) 12. Increased retrieval time 13. Coagulopathy or disseminated intravascular coagulation (DIC) 14. Advanced age   CT   1. Multilobar or bihemispheric injury 2. Ventricular injury with hemorrhage 3. Diffuse fragmentation 4. Missile passing through the geographic center of the brain (i.e., involving the thalamus and basal ganglia) 5. An area 4 cm above the dorsum sellae was described as the zona fatalis [16] 6. Trajectory crossing the x, y and z planes 7. Midline shift [10 mm on CT (Caveat: Kim et al. [16] found midline shift was associated with better outcome presumably because one hemisphere is traversed rather than both hemispheres) 8. Compressed or obliterated basal cisterns 9. Large intracerebral hemorrhage 10. Subarachnoid hemorrhage (SAH) 11. Large volume of contused brain 12. Posterior fossa wound with brainstem involvement 13. ‘Tram track sign’ hemorrhage on either side of a dark center track in a perforating injury |
